# Menstrual pattern and self‐reported abnormal uterine bleeding in Brazilian adolescents: A multicenter cross‐sectional study

**DOI:** 10.1002/ijgo.70243

**Published:** 2025-05-31

**Authors:** Renan Massao Nakamura, Gabriela Pravatta Rezende, Daniela Angerame Yela, Cristina Laguna Benetti‐Pinto

**Affiliations:** ^1^ School of Medicine, University of Campinas (UNICAMP) Campinas Brazil; ^2^ Department of Obstetrics and Gynecology School of Medical Sciences, State University of Campinas Campinas São Paulo Brazil

**Keywords:** abnormal menstrual bleeding, adolescence, heavy menstrual bleeding, menstruation, quality of life, reproductive health

## Abstract

**Objective:**

This study describes the menstrual patterns and prevalence of heavy menstrual bleeding among Brazilian adolescents, compared to adult women, and assesses the impact of heavy menstrual bleeding on their quality of life.

**Methodology:**

This multicentric cross‐sectional study was conducted over 2021 and 2022 and included 1761 women, of whom 195 were adolescents of reproductive age (≤19 years old). Sociodemographic data, menstrual cycle characteristics, and self‐perception of the effects on quality of life of menstrual bleeding were collected through questionnaires.

**Results:**

The mean age of adolescents was 16.70 ± 1.99 years; 18.5% of participants were classified as overweight or obese. The mean age of menarche was 11.88 ± 1.16 years old. Heavy menstrual bleeding was reported by 20.51% of the adolescents, compared to 32.76% of the adults (*P* < 0.001), and 38.53% had been diagnosed with anemia. A significant percentage of adolescents (61%) reported an impact of menstrual cycles on their quality of life, with 74.9% experiencing dysmenorrhea. Among adolescents with heavy menstrual bleeding, 57.5% reported menstrual cycle irregularity; among adolescents without heavy menstrual bleeding, 16.1% reported menstrual cycle irregularity.

**Conclusion:**

The prevalence of heavy menstrual bleeding was 20.5% among Brazilian adolescents. Most of them had irregular cycles, with periods of amenorrhea in 38%. A large percentage of them reported worsening in their quality of life during menstrual bleeding, and 38.5% had already experienced anemia throughout their life.

## 1 INTRODUCTION

Adolescence is defined as a biopsychosocial period that comprises, according to the World Health Organization, the second decade of life; that is, from 10 to 19 years of age.^1^ Adolescents often have menstrual irregularities that persist into adulthood or disappear with age. One of the transient causes is the immaturity of the hypothalamic–pituitary–ovarian axis. Thus, the first years of reproductive life, which begins with the first menstrual period, demonstrating ovarian function, are associated with menstrual cycle‐related complaints, including abnormal uterine bleeding (AUB). The age of menarche is an important marker of the onset of female puberty and can vary greatly between different regions of the world, influenced by genetic, nutritional, socioeconomic and environmental factors. In South America, the average age of the first menstruation is 12 years.^2^ The prevalence rates of AUB in adolescents range from 4% to 63% and are frequently associated with physical and psychological problems, including dysmenorrhea, pelvic pain, and mood and sleep disorders.^1^, ^2^, ^3^ Adolescence is reported to be an age of vulnerability, and suffering from AUB has negative repercussions on overall health and quality of life, leading to school absenteeism, social limitations, and greater need for medical visits and treatment.^3^, ^4^

Although non‐structural alterations, especially ovulatory dysfunctions, are the more prevalent causes of AUB among adolescents,^5^, ^6^ some causes have long‐term consequences, such as coagulation disorders. Despite the severity of this condition, 85% of AUB cases in adolescents occur in the first two years after menarche and only a small number are the result of coagulation factor deficiency (approximately 3.8%).^5^ Other authors evaluated 2770 adolescents and also indicated ovarian dysfunction as the most frequent cause of AUB, while coagulation disorders and platelet disorders accounted for 19.4 and 6.23% of AUB cases, respectively. However, the percentage of cases without a defined cause was high, at 45.9%.^7^

The prevalence of AUB in the Brazilian general population was only recently assessed, showing that 31.4% of Brazilian women of reproductive age have AUB, although there is no specific data for adolescents.^8^ According to the latest Brazilian CENSUS (2022),^9^ there are approximately 14 million female adolescents, accounting for approximately 7% of the country's total population. The discrepancies in the literature associated with the scarcity of specific data for this age group, both regarding the menstrual pattern and the adolescents' self‐perception of their menstrual cycle and its impact on their quality of life, motivated the present study. The aim is to describe the menstrual patterns of Brazilian adolescents and assess the prevalence of AUB in adolescents compared to adult women and the impact of AUB on their quality of life.

## 2 METHODS

This study is a subanalysis of a multicenter cross‐sectional study with the participation of eight representative centers from the five official geographic regions of Brazil, coordinated by the Department of Obstetrics and Gynecology of the School of Medical Sciences at the University of Campinas (UNICAMP).^8^

Women experiencing spontaneous menarche and women up to 55 years of age in general medical treatment in outpatient clinics or accompanying family members to medical appointments were included in the main study. Women treated in specialized outpatient clinics for abnormal bleeding were not included in the study, to avoid selection bias. Women in the pregnancy–puerperal cycle, those who had undergone hysterectomy or bilateral oophorectomy, those diagnosed with disorders that prevented them from adequately understanding the questions asked, and postmenopausal women were excluded.

**FIGURE 1:**
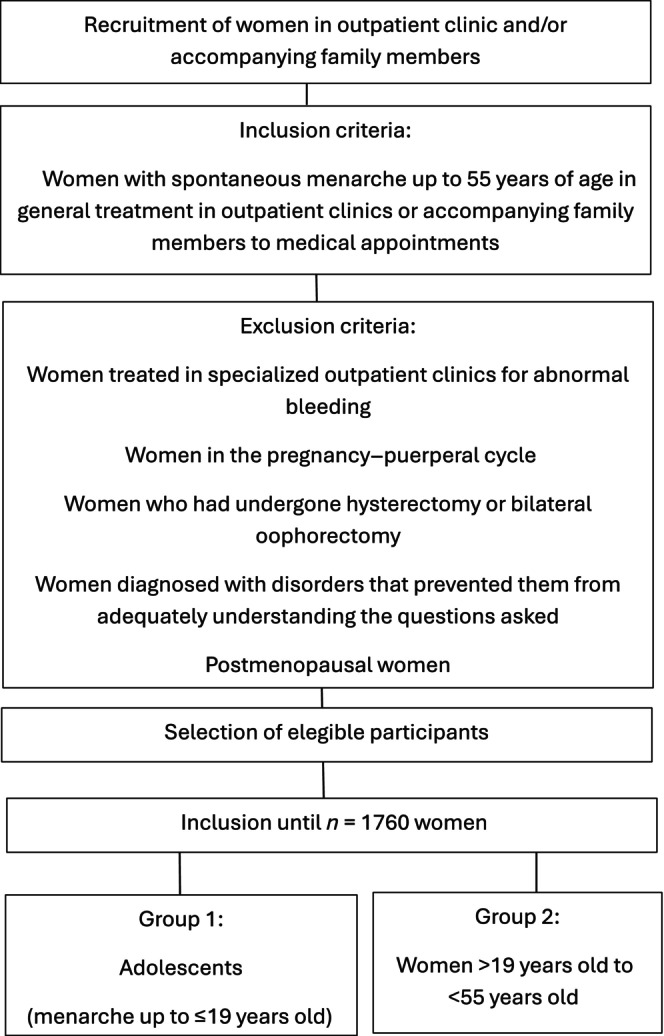
Sample selection methodology.

The inclusion of subjects for data collection was extended until the calculated sample (*N*) of 1760 women of different age was reached. Women were divided into two groups for the analysis: (i) adolescent women experiencing their first period and women aged up to 19 years of age; and (ii) adult women in their reproductive period, aged >19 years and <55 years. This study focused on adolescent women (Figure 1).

All women answered a questionnaire in person to elicit sociodemographic data (i.e., age, socioeconomic profile, obstetric history, self‐declared ethnicity, and years of education), characteristics of the menstrual cycle (i.e., self‐perception of menstrual bleeding, characterized as normal or heavy bleeding; duration of the menstrual cycle; and days of menstrual flow), symptoms concomitant with the menstrual period, and the need to use one or more combined sanitary products (e.g., external pads and regular tampons and/or a menstrual cup). All women were also asked to self‐assess the effects of their menstrual period on their quality of life. They were asked to respond using the visual analogue scale, ranging from 0 to 10, where zero means no impact and 10 represents the worst possible impact. The parameters defined by The International Federation of Gynecology and Obstetrics (FIGO) were used to define the normal parameters of the menstrual cycle.^3^ The country's official parameters, defined by the Brazilian Association of Research Companies, called Critério Brasil,^10^ were used in the socioeconomic analysis. According the socioeconomics parameters defined by Critério Brasil, the stratification is classified from A to E; were represents the best stratification, and E represents the lowest and worst socioeconomic stratum.

### 2.1 Statistical analysis

The sample size was calculated using the estimation formula based on the estimated prevalence of AUB in a literature study by Barcelos et al.^11^ The alpha significance level was set at 5% and the sampling error at 3%, and a minimum sample size of 1760 women was calculated.^8^ Categorical variables were described using absolute and percentage frequency values (*n*/%) and numerical variables using mean and standard deviation values. Continuous variables were described using mean and standard deviation and evaluated using the Intercooled Stata 13.0 program. The significance level adopted was 5%. The SAS for Windows, version 9.4, was used for statistical analysis (SAS Institute Inc., 2002–2012, Cary, NC, USA).

### 2.2 Ethical aspects

This study was approved by the ethics committee of the coordinating center and of each participating center (CAAE 40654720.0.1001.5404). Women or the legal guardians of minors signed the informed consent form and the assent form, respectively.

The study was developed with the support of the Brazilian Federation of Gynecology and Obstetrics Associations (FEBRASGO) and with financial support from Bayer S.A. The sponsors had no influence or participation in the development or analysis of data and interpretation of the results or in the writing of this scientific article.

## 3 RESULTS

A total of 1928 women from all five regions of Brazil were included in the study. After excluding menopausal women, 1761 women of reproductive age were analyzed. Among them, 195 (11.07%) were adolescents who had already had menarche (≤19 years of age), with 15.9% from the North region, 14.36% from the Northeast region, 13.85% from the Central‐West region, 41.03% from the Southeast region, and 14.87% from the South region, representing all official regions of the country. The mean age and body mass index of adolescents was 16.70 ± 1.99 years and 22.23 ± 4.06 kg/m^2^, respectively, and 18.5% were overweight or obese. There was no difference in the socioeconomic distribution between adolescents and adult women (*P* = 0.290) (Table 1).

**TABLE 1:** Sociodemographic data of included adolescents and reproductive period adult women.

	Adolescents (≤19 years) (*N* = 195)	Adult women (>19 years and <55 years) (*N* = 1566)	*P*
Mean age in years (SD)	16.70 (1.99)	35.49 (9.3)	
Mean BMI kg/m^2^ (SD)	22.23 (4.06)	25.7 (5.10)	*P* < 0.001^a^
Ethnicity			*P* 0.645^b^
White (%)	120 (61.5)	916 (58.5)	
Non‐white (%)	75 (38.5)	650 (41.5)	
Social stratification			*P* 0.290^a^
A (%)	50 (25.6)	317 (20.3)	
B1 (%)	32 (16.4)	241 (15.4)	
B2 (%)	36 (18.5)	368 (23.5)	
C1 (%)	25 (12.8)	235 (15.0)	
C2 (%)	25 (12.8)	22 (14.4)	
D‐E (%)	27 (13.8)	179 (11.4)	
Pregnancy (%)	8 (4.1)	1066 (61.5)	*P* < 0.001^a^
Years of education (SD)	11.0 (2.8)	14.9 (5.4)	*P* < 0.001^a^
Age at menarche (SD)	11.88 (1.16)	12.46 (1.54)	

Abbreviation: SD, standard deviation.

^a^ *χ* ^2^‐test.

^b^ Fisher's exact test.

**FIGURE 2:**
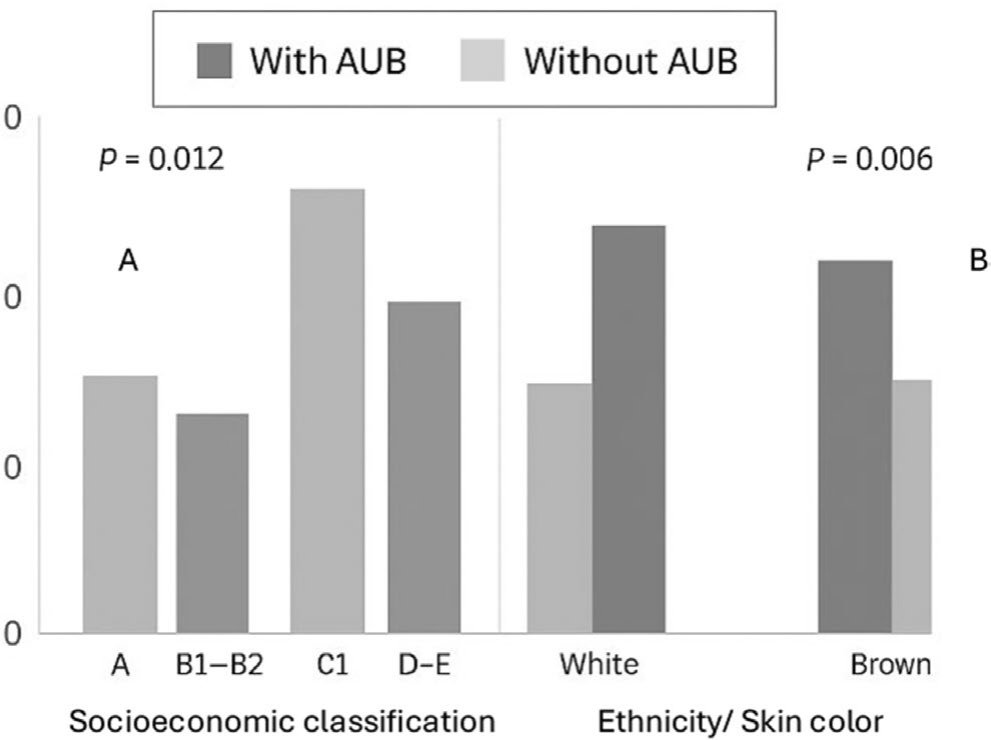
Distribution of Brazilian adolescents with and without abnormal uterine bleeding (AUB) according to socioeconomic classification and self‐reported skin color. A = *χ* ^2^‐test. B = Fisher's exact test.

Among adolescents, 4.10% had already been pregnant. It is noteworthy that 6.7% of the adolescents already considered themselves the head of the family; that is, they were economically responsible for the family. According to the self‐perception of adolescents, classified as having or not AUB, adolescents from lower social strata and non‐whites (brown and black) had a higher prevalence of AUB (Table 1; Figure 2).

### 3.1 Menstrual cycle characteristics

**FIGURE 3:**
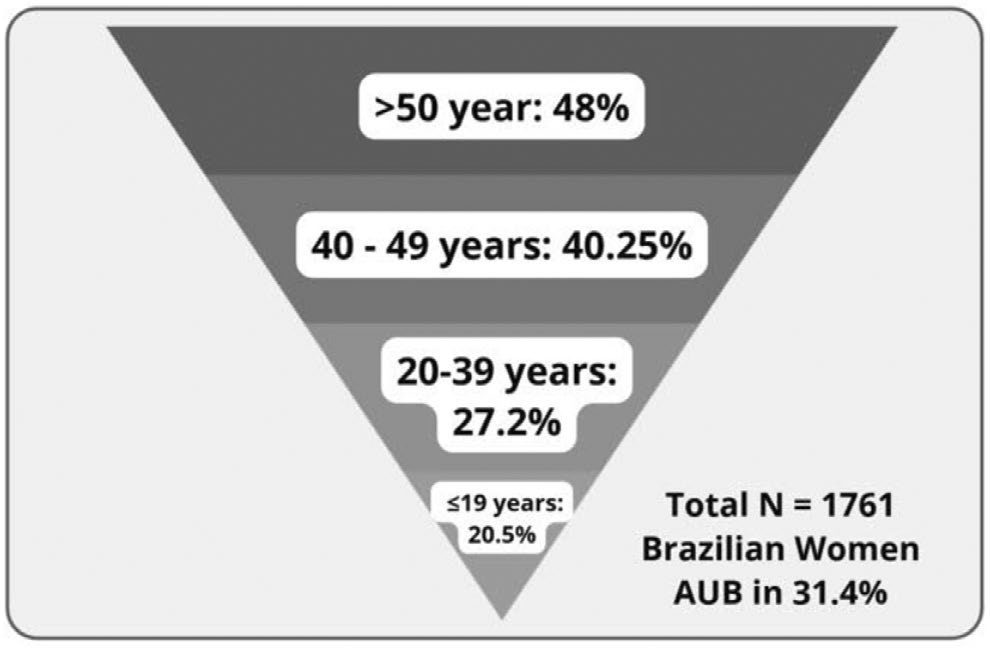
Prevalence of abnormal uterine bleeding (AUB) in the total population of Brazilian women in the reproductive period (*N* = 1761) among adolescents (*N* = 195) and among adult women (*N* = 1566) in different age groups.

Menarche occurred at 11.88 ± 1.16 years of age in the Brazilian adolescent population. The mean number of days of the menstrual interval was 29.24 ± 18.42, and the mean number of days of menstrual flow was 5.73 ± 2.36. The characterization of the menstrual cycle (Table 2) in terms of its duration or the number of days of bleeding did not differ between adolescents and adult women. Considering the normality parameters defined by FIGO,^3^ approximately 75% of the adolescents had menstrual cycles of normal duration (24–38 days), 92% had normal menstrual flow duration (up to 8 days), and 80% considered they did not have abnormal bleeding. In the total population of Brazilian women, the self‐perception of AUB was 31.4%. When divided into adolescents and adult women, 20.51% of Brazilian adolescents reported self‐perception of AUB, a lower number than that reported by adult women (*P* < 0.001; Table 2, Figure 3).

**TABLE 2:** Menstrual cycle characteristics of Brazilian adolescents and reproductive period adult women.

	Adolescents (≤19 years) (*N* = 195)	Adult women (>19 years) (*N* = 1566)	
Interval between periods			*P* = 0.763^a^
<24 days	32 (16.4)	291 (18.8)	
24–38 days	146 (74.9)	1113 (71.8)	
>38 days	16 (8.20)	132 (8.5)	
Menstrual flow			*P* = 0.007^a^
Up to 8 days	179 (91.8)	1426 (91.1)	
>8 days	15 (7.7)	125 (8.0)	
Self‐perception of bleeding			*P* < 0.001^a^
Normal	155 (79.5)	1053 (67.2)	
AUB	40 (20.5)	513 (32.8)	

Abbreviation: AUB, abnormal uterine bleeding.

^a^ *χ* ^2^‐test.

Considering only the group of adolescents with a self‐perception of AUB (20.5%), more changes were observed in the objective parameters for assessing AUB, with a higher frequency of cycles lasting fewer than 24 days or more than 38 days (*P* < 0.001) and flow lasting more than 8 days (*P* < 0.001) compared to adolescents without AUB, in addition to a higher frequency of reports of amenorrhea (Table 3).

**TABLE 3:** Comparative data between adolescents with self‐perception of AUB and without AUB.

	Adolescent with AUB (*N* = 40, 20.5%)	Adolescent without AUB (*N* = 155, 79.5%)	
Interval between periods			
<24 days	11 (27.5%)	21 (13.5%)	*P* < 0.001^a^
24–38 days	16 (40.0%)	130 (83.9%)	
>38 days	12 (30.0%)	4 (2.6%)	
Amenorrhea			
No	25 (62.5%)	132 (85.2%)	*P* = 0.001^b^
Yes	15 (37.5%)	23 (14.8%)	
Menstrual flow			
<4 days	2 (5.0%)	16 (10.3%)	*P* < 0.001^a^
4–8 days	25 (62.5%)	136 (87.7%)	
>8 days	12 (30.0%)	3 (1.9%)	
Anemia			
No	24 (61.5%)	110 (79.7%)	*P* = 0.002^b^
Yes	15 (38.5%)	28 (20.3%)	
Unable to answer	1 (2.5%)	17 (10.9%)	
Anemia treatment			
Oral iron	10 (25.6%)	17 (12.3%)	*P* = 0.024^a^
Intravenous iron	01 (0.6%)		

*Note*: Some adolescents were unable to answer.

^a^ *χ* ^2^‐test.

^b^ Fisher's exact test.

**FIGURE 4:**
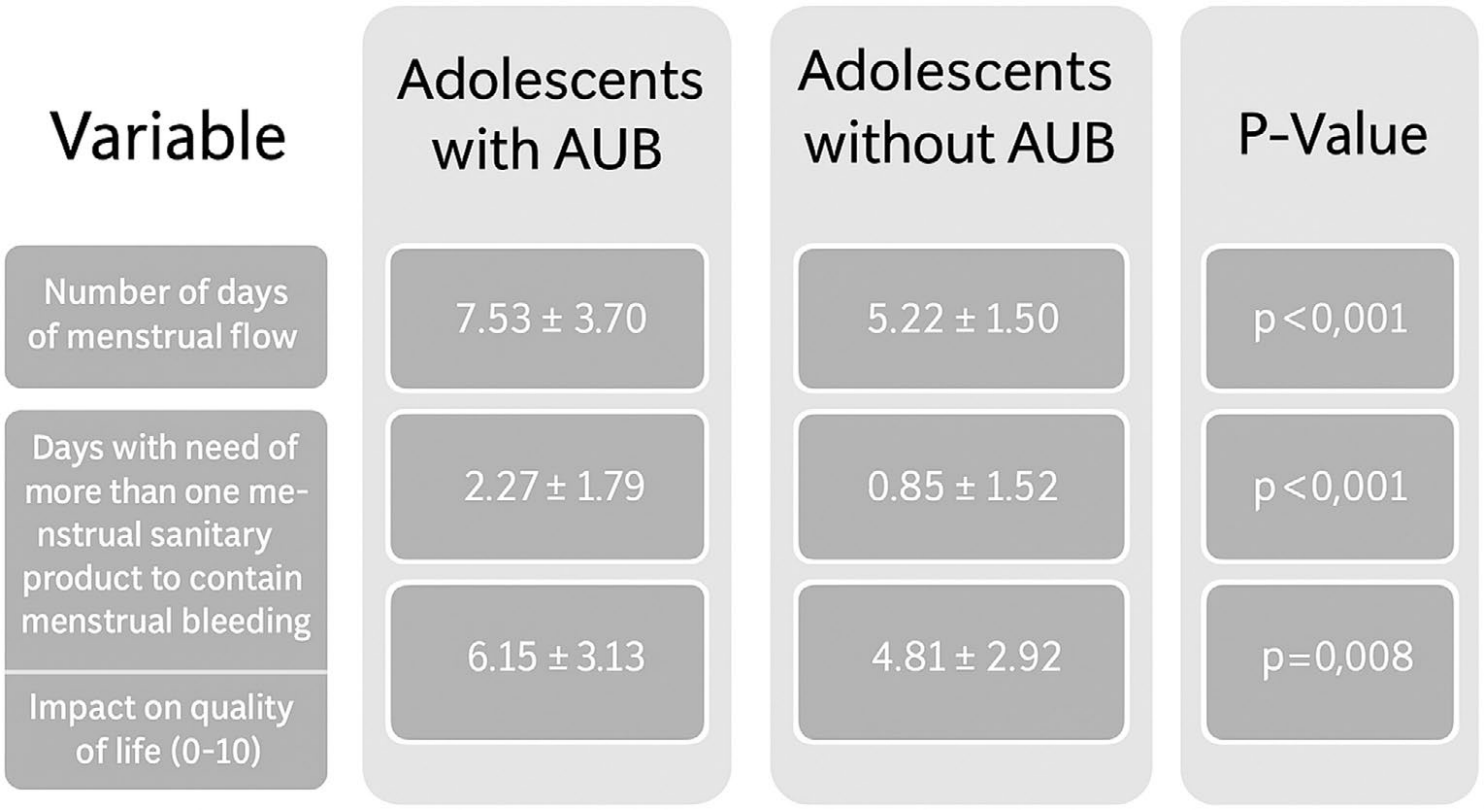
Objective data comparing adolescents with and without self‐reported abnormal uterine bleeding (AUB).

Adolescents with AUB had a higher frequency of anemia diagnosis (38.5%) and more needed iron replacement treatment (25.6%) than adolescents without heavy bleeding (*P* = 0.024). Intravenous iron replacement was also necessary for one adolescent (0.6%). Adolescents with AUB also needed to use more than one combined menstrual sanitary product (at the same time) for more days than those without AUB. When asked about the impact of the menstrual period on their quality of life, assessed according to the analog scale from 0 (no impact) to 10 (worst impact), 61% of the adolescents reported that their menstrual period worsens their quality of life; the greatest and worst impact was found among those with AUB (Figure 4). The presence of dysmenorrhea was reported by 74.9% of adolescents.

## 4 DISCUSSION

This study was conducted with the inclusion of women from all regions of Brazil. A total of 1761 women were evaluated, of which approximately 200 were adolescents. The prevalence of AUB among adolescents was 20.5%. Brazilian adolescents with AUB experience irregular cycles more frequently than adolescents without AUB and longer menstrual cycles. They are also diagnosed with anemia more frequently, in addition to showing greater impairment in their quality of life during the menstrual period compared to adolescents without AUB.

This study is a secondary analysis of a study that assessed the prevalence of AUB in the Brazilian population.^9^ The adolescent population assessed (from menarche to 19 years) had a regional distribution similar to the percentage of Brazilian women aged 10–19 years obtained in the 2022 CENSUS.^9^ The highest concentration of adolescents was observed in the Southeast region (41.03%), in contrast to the other regions, which are less populated.

For some authors, the most common complaint among adolescents who need to seek medical care for health issues is the presence of AUB. However, data indicating the prevalence of AUB in adolescents^12^ are highly variable. Also through a population study, Friberg et al.^13^ reported a prevalence of 37%, while a recent literature review by Pouraliroudbaneh et al. indicated rates ranging from 4% to 63%.^5^, ^13^, ^14^ These discrepancies, linked to different methodologies and populations, highlight the need for more evidence, perhaps with specific populations or larger samples that would allow its use in health actions targeted at this age group.

To date, there are few publications evaluating adolescents with AUB, meaning the true prevalence was still unknown.Our results in Brazil, based on adolescents from across the country, revealed a prevalence of 20.5% Abnormal Uterine Bleeding (AUB) among them. Adolescence is a period of variability in the menstrual cycle, and the immaturity of the reproductive system, in a physiological way, can affect the behavior of the menstrual cycle.^14^ Our results showed that adolescents with AUB have an irregular menstrual pattern more frequently; considering standards established by FIGO,^3^ as approximately 60% report short or long cycles and 38% have periods of amenorrhea, numbers significantly higher than those of adolescents without AUB, since 84% report regular menstrual cycles (24 to 38 days).

A systematic review indicated that adolescents can have various causes of bleeding, including those persisting throughout life, such as coagulation disorders.^7^ Adolescents with AUB since their first menstruation and also diagnosed with anemia should be investigated for the presence of coagulopathies.^15^, ^16^ The diagnosis and education of young women with coagulation disorders and AUB allows them to be empowered in the face of situations that can lead to higher rates of isolation, anxiety, and discomfort.^17^ However, these authors also showed ovulatory dysfunction as the most frequent cause of AUB among adolescents. Our data is consistent with the current literature, considering that Brazilian adolescents with AUB had longer cycles and periods of amenorrhea. Considering that anovulation is a disorder directly associated with this period of reproductive life, it is advisable that health professionals act to prevent its repercussions for the health of these young women. Several studies unequivocally indicate the association between AUB and anemia, requiring medical intervention and treatment, as well as clinical complaints associated with the condition.^4^, ^8^, ^18^, ^19^ We found previously diagnosed anemia in almost 40% of adolescents. This high prevalence of anemia highlights the need for nutritional interventions and health programs aimed at treating this condition, preventing its persistence, or even preventing new anemic conditions, especially considering the long‐term health implications. Our study also showed that adolescents with AUB had impacts on their quality of life due to the more frequent use of more than one absorbent sanitary product at the same time, which is also highlighted in the published literature.^18^, ^19^, ^20^

There is also evidence that AUB is associated with more diagnoses of depression and worsening quality of life, with an effect on participation in sports, school absenteeism, and social isolation.^17^, ^18^, ^19^, ^20^, ^21^, ^22^ The adolescents we assessed reported a greater reduction in their quality of life than those of the same age who did not have AUB. The impact of AUB on Quality of life reported by the world population^17^, ^21^, ^22^ has motivated the growth of a movement to raise awareness of the effects of the menstrual period, which emerged to draw attention to the difficulties in accessing infrastructure, healthcare and sanitary products of better quality to strengthen public policies that provide a safe and less negative menstrual period. Objective prevalence data in the present study combined with data showing impairment of health in the overall sense and quality of life are decisive for the implementation of actions aimed at offering menstrual dignity. Adolescents, considered to be at a more vulnerable age, should be the focus of these actions.

The main strength of this study is being the first to assess the Brazilian population in a multicentric manner in all its regions, considering its socioeconomic disparities. Furthermore, the prevalence of AUB among adolescents in Brazil was estimated in a pioneering way. These results may be used to alert health managers of the need for differentiated care for this age group. However, the study has limitations: its cross‐sectional design, in which not only the population of young people up to 19 years of age was initially targeted, does not allow inferences of cause and effect, only delimiting and describing the menstrual patterns of Brazilian adolescents.

Nevertheless, our results provide a comprehensive view of the sociodemographic, clinical, and menstrual characteristics of Brazilian adolescents. The significant differences between adolescents and adult women in terms of reproductive health and the perception of the menstrual cycle underscore the need for public health policies addressing these issues in a targeted and culturally sensitive manner. Educational and health programs aimed at adolescents should be expanded to ensure young women's access to the information and resources necessary for effective care.

## 5 CONCLUSION

The prevalence of AUB is 20.5% among Brazilian adolescents. Most have irregular and/or long menstrual cycles, and 38% have periods of amenorrhea. A large proportion (24.3%) have already had anemia and needed treatment, and 61% reported a worsening of their quality of life during their menstrual period. Therefore, AUB affects a significant proportion of Brazilian adolescents, with considerable impact on menstrual regularity, anemia history, and quality of life. These findings underscore the need for greater attention to adolescent health, particularly in the early identification and management of AUB. Future research is warranted to better understand the underlying factors, long‐term consequences, and most effective interventions to improve outcomes for this population.

## AUTHOR CONTRIBUTIONS

All the authors participated actively in the study, as follows: All the authors participated actively in the study, as follows: Conception and design – RMN, GPR, DAY, CLBP; Acquisition of data – RMN, GPR; Analysis and interpretation of data – RMN, GPR, DAY, CLBP; Drafting the article – RMN, GPR, DAY, CLBP; Revising it for intellectual content – GPR, DAY, CLBP; Final approval of the completed article – RMN, GPR, DAY, CLBP. The authors are responsible for the decision to submit the manuscript for publication. The authors do not have any potential conflict of interest.

## FUNDING INFORMATION

This was an investigator‐initiated study with financial support from Bayer S.A. The funding sources had no role in the study conduct, analysis, interpretation of data, or decision to publish the results. The study was carried out with the support of the Brazilian Federation of Gynecology and Obstetrics (FEBRASGO).

## CONFLICT OF INTEREST STATEMENT

The authors declare no conflicts of interest.

## Data Availability

The data are available at Unicamp Repository: Gabriela Pravatta Rezende Antoniassi, 2024, “Menstrual pattern and prevalence of abnormal uterine bleeding in Brazilian adolescents –A multicenter cross‐sectional study,” https://doi.org/10.25824/redu/GOA9RV, Repositório de Dados de Pesquisa da Unicamp, draft version.
